# Phylogenetic Analysis of the Most Common Chicken Coccidia, *Eimeria acervulina*, Isolated From Commercial Poultry Farms in Bangladesh

**DOI:** 10.1002/vms3.71309

**Published:** 2026-11-03

**Authors:** Bimal Chandra Karmakar, Nusrat Nowrin Shohana, Subir Sarker, Anita Rani Dey, Anisuzzaman, Sharmin Aqter Rony, Shanaz Parvin, Saykat Kumar Debnath, Shirin Akter, Mohammad Zahangir Alam

**Affiliations:** ^1^ Department of Parasitology Faculty of Veterinary Science Bangladesh Agricultural University Mymensingh Bangladesh; ^2^ Department of Biomedical Sciences and Molecular Biology College of Medicine and Dentistry James Cook University Townsville Queensland Australia; ^3^ Department of Para‐Clinical Courses, Faculty of Veterinary and Animal Sciences Gono Bishwabidyalay Savar Dhaka Bangladesh

**Keywords:** Bangladesh, chicken coccidia, *E. acervulina*, ITS‐1, phylogenetic analysis

## Abstract

**Background:**

*Eimeria acervulina* is the predominant species of coccidia infecting chickens worldwide, leading to significant production losses. The study explores molecular detection and phylogenetic relationships of *E. acervulina* in poultry farms of Bangladesh.

**Methods:**

A total of 206 microscopically positive faecal samples were processed for PCR amplification. Oocysts were isolated using flotation techniques following microscopic examination. After recovering the genomic DNA, PCR amplification was performed using species‐specific primers focusing on the internal transcribed spacer‐1 (ITS‐1) region. A subset of 36 *E. acervulina*‐positive samples was randomly sequenced and analysed for genetic diversity and phylogenetic relationships.

**Results:**

A total of 198 (96.11%) *E. acervulina* samples with a specific 321‐bp amplicon were detected. Sequence analysis identified 15 distinct genotypes and seven single‐nucleotide substitutions, indicating intra‐species genetic variation. Nucleotide diversity (0.00717) and genotype diversity (0.911) suggested a genetically diverse population with closely related variants. The studied genotypes of *E. acervulina* formed a cluster with the previously identified reference sequence retrieved from GenBank with strong bootstrap support (98%). Similarly, other *Eimeria* species formed distinct clades independent of geographic origin. Among the studied sequences, two major clusters were observed, with no clear regional structuring.

**Conclusion:**

This study provides the first molecular characterization of *E. acervulina* from commercial poultry farms in Bangladesh. The findings reveal measurable genetic diversity of *E. acervulina* circulating in Bangladesh, providing baseline data for epidemiological understanding and control strategies for avian coccidiosis.

## Graphical abstract

The ITS‐1 PCR assay detected 96.11% *E. acervulina* in coccidia‐positive faecal samples from commercial poultry farms. The sequence analysis revealed 15 genotypes and seven single‐nucleotide substitutions, indicating significant genetic diversity. This is the first report of molecular characterization of *E. acervulina* in Bangladesh.

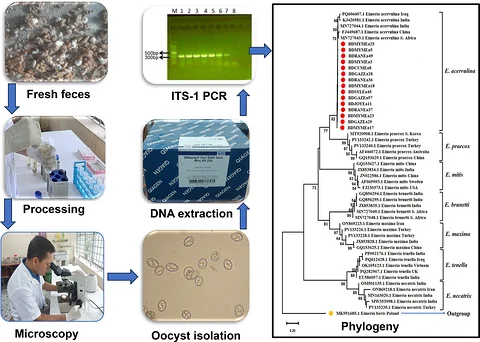

## 1 Introduction

The poultry industry stands as one of the most progressive agricultural fields globally and plays a critical role in food security and nutrition. Demand for poultry products is predicted to continue rising during the coming days (Mottet and Tempio 2017). However, productivity is constrained by various infectious agents, including those caused by protozoan parasites. Among protozoa, *Eimeria* species are responsible for a commercially important disease, coccidiosis, where *Eimeria acervulina*, *Eimeria tenella*, *Eimeria necatrix*, *Eimeria maxima*, *Eimeria brunetti*, *Eimeria mitis* and *Eimeria praecox* are commonly reported and infect specific regions of the gastrointestinal tract (Soulsby 1982). For instance, *E. acervulina* primarily localizes in the upper gastrointestinal tract, specifically the duodenum and the proximal jejunum (Campos et al. 2024). Infection involves repeated invasion and destruction of entero‐epithelial cells, resulting in intestinal inflammation and, in severe cases, haemorrhage. In commercial production systems, contaminated feed, water or litter serves as a rapid route of disease transmission (Conway and McKenzie 2007). The higher prevalence of *E. acervulina* in the litter sample of commercial poultry farms in Bangladesh was reported (Alam et al. 2020). It causes malabsorptive coccidiosis, a disease characterized by mucoid enteritis, where inflammation occurs in the intestinal tract and accumulation of mucus may lead to weight loss (Blake and Tomley 2014). *Eimeria* lesions can predispose chickens to *Clostridium perfringens* infection, resulting in mortality from necrotic enteritis (Moore 2016). Consequently, coccidiosis imposes a substantial economic burden, with global losses estimated at approximately US$14 billion annually, including impacts in Bangladesh (Blake et al. 2020; Rony et al. 2021). Coccidiosis is widely reported in poultry farms across America, Europe and Asia (Fornace et al. 2013). In Bangladesh, multiple studies using conventional and molecular methods have identified several *Eimeria* species (Karim and Begum 1994; Karim and Trees 1990), showing bird‐level prevalence of 22%–34.48% (Alam et al. 2020, 2021). Given its high prevalence and its use as a model organism for homologous and heterologous vaccine studies (Tang et al. 2018), precise identification and genetic lineage‐related characterization are essential.

Traditional diagnostic methods, including oocyst morphology, sporulation time and lesion scoring, are limited by morphological overlap among species and require considerable expertise, increasing the risk of misidentification (Long and Joyner 1984). Molecular approaches targeting internal transcribed spacer‐1 (ITS‐1) (Schnitzler et al. 1999), ITS‐2 (Lien et al. 2007) and the 5.8S rRNA gene (Molloy et al. 1998) provide reliable tools for species identification. As genetic diversity influences epidemiological trends, pathogenicity and control outcomes, phylogenetic analysis of *E. acervulina* is important for improving diagnostics and informing control strategies. Although substantial research has focused on *E. tenella* due to its pathogenicity and economic importance (Blake et al. 2015; Võ et al. 2021; Alam et al. 2022), *E. acervulina* has received comparatively minor attention. This is notable given its widespread occurrence and role in subclinical infections that reduce production efficiency. Therefore, this research focused on investigating the genetic diversity of *E. acervulina* isolates from Bangladesh using the ITS‐1 gene region.

## 2 Materials and Methods

### 2.1 Study Area and Poultry Farm Selection

A total of 350 fresh faecal samples were collected from seven major poultry‐producing regions of Bangladesh, such as Mymensingh, Cumilla, Barishal, Gazipur, Rangpur, Joypurhat and Sylhet. The sample size was calculated through the equation given by Thrusfield (1995). According to this formula, sample size (*n*) = *Z* ^2^[P(1 − P)]/d^2^. Here, *n* = sample size and *Z* = the standard normal deviate for the desired confidence level. For a standard 95% confidence interval, *Z* = 1.96. *P* = expected prevalence: 65% (*P* = 0.65), d = absolute precision: 5% = 0.05. So, *n* = 349.44 ≈ 350. Due to the low probability of *Eimeria* infection in birds below 15 days of age (Adhikari et al. 2008; Iqbal and Begum 2010), 15‐ to 60‐day‐old Sonali and Broiler farms, reared on floor‐rearing system, were purposively selected for sample collection.

### 2.2 Sample Collection and Oocyst Isolation

About 100 g of fresh faecal material was obtained from each farm. Samples positive for *Eimeria* oocysts upon microscopic examination were processed for isolation according to the procedure of Alam et al. (2020). Oocysts were concentrated using a flotation technique with saturated sodium chloride solution (specific gravity 1.18–1.20). Sporulation was induced by incubating oocysts in 2.5% potassium dichromate at 28°C for 24–48 h, followed by subsequent pellet formation through repeated centrifugation and removal of the supernatant. Among the microscopically positive samples, 206 samples were processed for molecular study. In this case, a minor modified McMaster technique (Soulsby 1982) was used to determine the number of oocysts per gram of faeces. Here, one gram of faeces contains ≥ 50 oocysts of any species of *Eimeria;* the poultry farm was considered for coccidia‐positive; otherwise, negative (Alemayehu et al. 2013). In our study, 10 oocysts in a McMaster chamber (≥ 500 OPG) were processed for molecular analysis.

### 2.3 Genomic DNA Extraction

Diluted oocyst samples were subjected to repeated centrifugation followed by resuspension in water to clean them (Alam et al. 2022). Then, the samples were allowed to undergo repeated freezing, thawing and sonication using a specialized tissue homogenizer to crush the oocysts. Finally, genomic DNA was extracted according to the manufacturer's protocol using the QIAamp stool DNA isolation kit (Qiagen, Germany). Eventually, the extracted DNA samples were kept at –20°C until further processing.

### 2.4 PCR Amplification and Sequencing of the ITS‐1 Gene

Species‐specific identification of *E. acervulina* was performed using a single PCR assay targeting the ITS‐1 region, as described by Schnitzler et al. (1999). The forward and reverse primers used were EaF (5′‐GGCTTGGATGATGTTTGCTG‐3′) and EaR (5′‐CGAACGCAATAACACACGCT‐3′), respectively. PCR amplification was carried out in a 25 µL reaction mixture containing 2 µL template DNA, 10 pmol of each primer, 3.0 mM MgCl_2_, 2.0 µL 10× PCR buffer, 200 µM of each dNTP and 0.4 U Taq DNA polymerase. The thermal cycling conditions included an initial denaturation at 95°C for 5 min, followed by 35 cycles of 95°C for 30 s, 60°C for 30 s and 72°C for 1 min, with a final extension at 72°C for 10 min. PCR products were visualized on a 2% agarose gels to confirm the expected amplicon size. A subset of 36 positive samples was purified using QIAquick kits (Qiagen) and sequenced on an automated sequencer using forward and reverse primers.

### 2.5 Phylogenetic and Sequence Analysis

ITS‐1 sequences were aligned using ClustalW implemented in MEGA software. Besides, nucleotide diversity, genotype diversity and average number of nucleotide differences were calculated using DnaSP v5.1 (Rozas 2009). Pairwise sequence identity (%) was determined using BioEdit (Hall 1999) through comparison with previously published sequences. Phylogenetic relationships were inferred using the maximum likelihood (ML) method based on the Tamura–Nei model (Tamura et al. 2013). Node support was evaluated using 1000 bootstrap replicates, and a 50% cut‐off was applied to generate the consensus tree.

## 3 Results

### 3.1 Species Determination and Genotyping of *E. acervulina*

Out of 206 coccidia‐positive faecal specimens obtained from commercial poultry farms across major poultry‐producing regions of Bangladesh, PCR amplification targeting the ITS‐1 region confirmed 198 samples (96.11%) as *E. acervulina* with a single species‐specific amplicon size of 321 bp (Figure 1). To investigate genetic variation, 36 representative PCR‐positive samples were selected for sequencing. The sequences showed strong homogeneity (99.20%–100%) with previously reported *E. acervulina* sequences through a BLASTn search, confirming accurate species identification.

**FIGURE 1:**
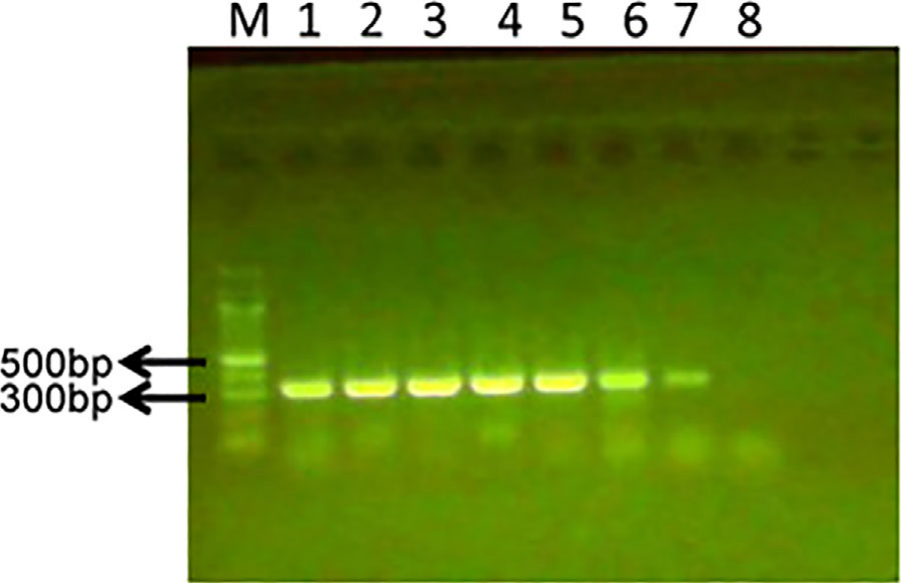
Agarose gel electrophoresis of the PCR products. Amplification of ∼321 bp ITS‐1 gene for *E. acervulina* represented on a 2% agarose gel; M, 100 bp marker; 1, positive control; 2–7, ITS‐1 gene product from samples; 8, negative control. In lane 7, the faint band indicates a low DNA concentration in the PCR product, likely due to a lower number of oocysts in the sample.

Despite this overall similarity, notable intra‐species variation was observed. Analysis of ITS‐1 sequences identified 15 distinct genotypes among the 36 isolates, indicating substantial genetic heterogeneity. However, these distinct genotypes were not correlated with the samples collected from different age and bird groups. Pairwise sequence identity ranged from 98.38% to 100% when compared both within study isolates and against reference sequences (GenBank accession numbers MN727044.1 and FJ449688.1). Alignment with the reference sequences (MN727044.1 and PQ282965.1) revealed seven single‐nucleotide substitutions at positions 99, 119, 122, 151, 160, 174 and 264. These substitutions included four transversions and three transitions (Table 1). Population genetic indices further characterized this variation. The nucleotide diversity (π) was relatively low (0.00717), whereas genotype diversity was high (0.911) (Table 2).

**TABLE 1:** Nucleotide variations and distribution of 15 genotypes of *E. acervulina* isolates with the reference sequences of *E. acervulina* of ITS‐1 gene.

Genotypes	Nucleotide positions							
	99	119	122	151	160	174	264	Number of isolates
MN727044.1 S. Africa	**A**	**G**	**C**	**G**	**A**	**G**	**T**	**—**
PQ282965.1 India	**A**	**G**	**C**	**G**	**A**	**G**	**T**	**—**
BDMYMEa3 (PZ235324)	.	T	.	.	.	.	.	3
BDMYMEa5 (PZ235325)	G	.	.	.	.	.	.	6
BDCUMEa8 (PZ235326)	.	T	.	.	.	.	G	5
BDJOYEa11 (PZ235327)	.	T	.	.	.	A	.	4
BDMYMEa17 (PZ235328)	.	T	T	T	.	A	.	1
BDMYMEa18 (PZ235329)	G	.	.	.	.	A	.	2
BDMYMEa23 (PZ235330)	.	T	.	.	.	A	.	1
BDMYMEa25 (PZ235331)	.	.	.	.	.	.	.	7
BDGAZEa28 (PZ235332)	G	.	.	.	.	.	G	1
BDGAZEa29 (PZ235333)	.	.	.	.	.	A	.	1
BDRANEa36 (PZ235334)	G	T	.	.	.	.	.	1
BDRANEa37 (PZ235335)	.	.	.	.	.	A	.	1
BDSYLEa45 (PZ235336)	.	.	.	.	.	A	.	1
BDRANEa49 (PZ235337)	G	T	.	.	T	.	.	1
BDGAZEa57 (PZ235338)	.	T	.	.	T	A	.	1
Total								**36**

*Note*: Dot (.) represents similar position with reference sequences.

**TABLE 2:** Genotype and nucleotide diversity of *E. acervulina* collected from poultry farms of different regions of Bangladesh.

Geographic regions	Number of sequences (*N*)	Number of genotypes (*n*)	Genotype diversity (Gd)	Nucleotide diversity (Nd)
Barishal	4	3	0.833	0.00469
Cumilla	4	2	0.500	0.00313
Gazipur	6	4	0.889	0.00833
Joypurhat	4	2	0.500	0.00156
Mymensingh	8	5	0.893	0.00826
Rangpur	5	5	1	0.00813
Sylhet	5	4	0.900	0.00563
Overall	36	15	0.911	0.00717

### 3.2 Phylogenetic Analysis of *E. acervulina*

Phylogenetic analysis was performed using 51 ITS‐1 sequences (15 study genotypes, 35 reference sequences and one outgroup) representing different *Eimeria* species from various geographic regions. The ML method based on the Tamura–Nei model was used, with *Eimeria bovis* (MK951685.1) serving as the outgroup. The study clearly placed all study‐derived sequences (designated with the prefix “BD” and further lebelled with PZ as individual accession number in the parentheses), such as BDMYMEa3 (PZ235324), BDMYMEa5 (PZ235325), BDCUMEa8 (PZ235326), BDJOYEa11 (PZ235327), BDMYMEa17 (PZ235328), BDMYMEa18 (PZ235329), BDMYMEa23 (PZ235330), BDMYMEa25 (PZ235331), BDGAZEa28 (PZ235332), BDGAZEa29 (PZ235333), BDRANEa36 (PZ235334), BDRANEa37 (PZ235335), BDSYLEa45 (PZ235336), BDRANEa49 (PZ235337) and BDGAZEa57 (PZ235338). The resulting phylogenetic tree demonstrated clear separation of *Eimeria* species into distinct clusters, supported by bootstrap values ranging from 73% to 98%. The isolates obtained in this study clustered strongly with previously reported *E. acervulina* sequences, with 82% bootstrap support, confirming their taxonomic identity (Figure 2).

**FIGURE 2:**
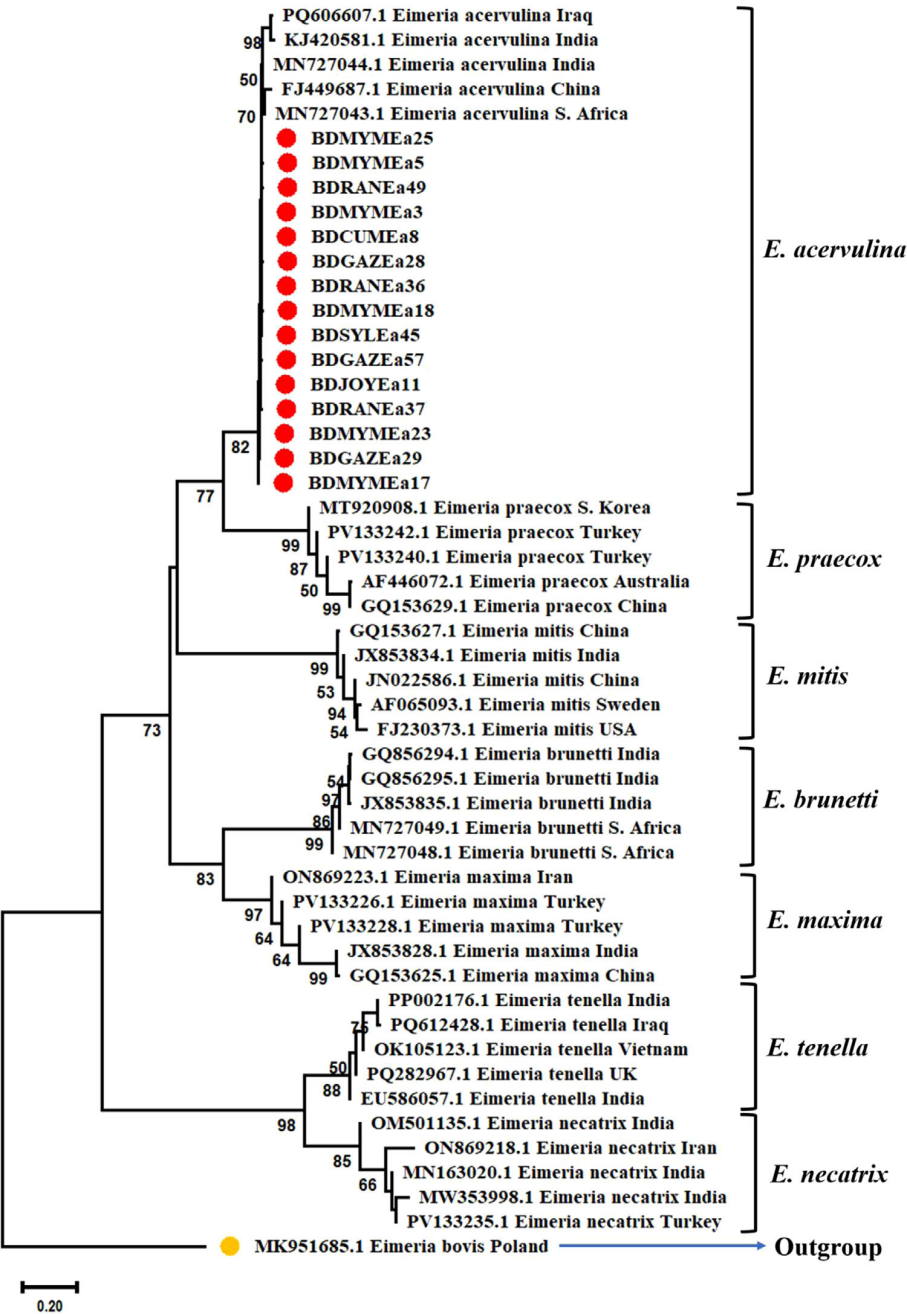
Maximum likelihood (ML) phylogeny of ITS‐1 gene sequences of *E. acervulina*. The analysis involved 51 nucleotide sequences (15 studied genotype sequences and 36 reference sequences, retrieved from the GenBank database). The percentage of replicate trees in which the associated taxa clustered together in the bootstrap test (1000 replicates) is shown next to the branches. Red bullets indicate studied genotype sequences.

A close evolutionary relationship was observed between *E. acervulina* and *E. praecox*, which formed a sister clade with a bootstrap value of 77%. In contrast, *E. tenella* and *E. necatrix* grouped in a separate clade with strong support (98%), consistent with their recognized biological and pathogenic similarities. Other species, including *E. maxima*, *E. mitis* and *E. brunetti*, generated a separate cluster within the phylogenetic tree. Notably, the clustering pattern did not correspond to geographic origin, as sequences from different continents were intermixed within species‐specific clades. Phylogenetic analysis restricted to the 36 ITS‐1 sequences from Bangladesh revealed two distinct clusters of *E. acervulina* (Figure 3). These clusters showed no clear association with sampling locations.

**FIGURE 3:**
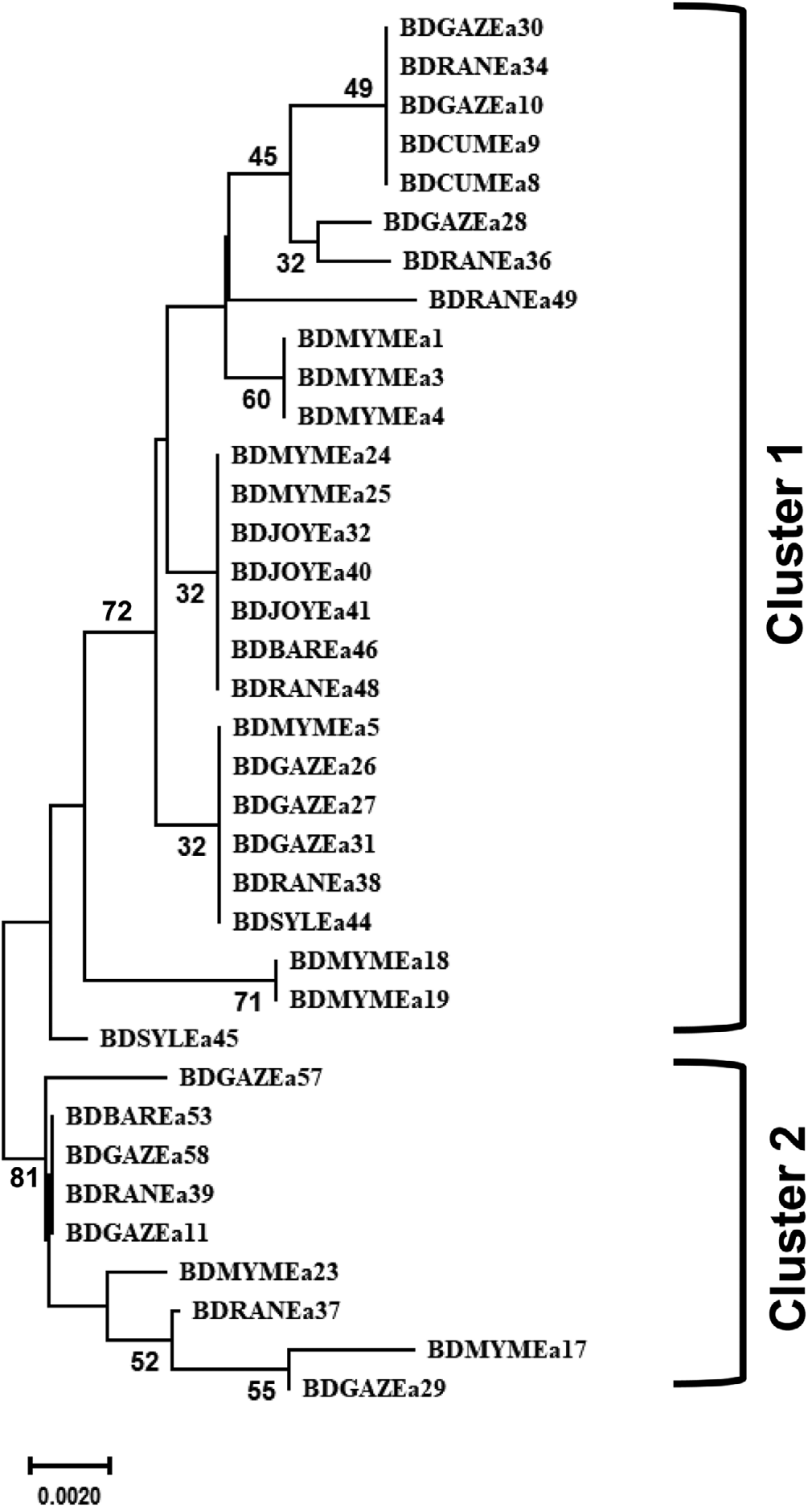
Maximum likelihood (ML) phylogeny showing the relationship among 36 *E. acervulina* sequences isolated from poultry farms in different regions of Bangladesh based on ITS‐1 sequences. The percentage of replicate trees in which the associated taxa clustered together in the bootstrap test (1000 replicates) is shown next to the branches.

## 4 Discussion

Coccidiosis caused by *Eimeria* spp. remains a critical obstacle to poultry production globally, including in Bangladesh (Ithurbide et al. 2026; Alam et al. 2020; Jenkins et al. 2017). Among the different species, *E. acervulina* is frequently reported as one of the most prevalent species and is primarily associated with subclinical infections that adversely affect production efficiency. The high prevalence observed in this study (96.11%) reinforces its epidemiological significance and suggests widespread distribution across commercial poultry systems in Bangladesh.

Morphological similarities among *Eimeria* species, together with variation associated with host factors and infection dynamics, can lead to misidentification by conventional microscopic examination (Vrba et al. 2010; Samuel Olufemi et al. 2020). Moreover, the frequent occurrence of mixed infections under field conditions further complicates accurate species identification (Kumar et al. 2014). These limitations highlight the importance of molecular approaches for reliable species‐level detection. In the present study, PCR amplification targeting the ITS‐1 region enabled specific identification of *E. acervulina*. Molecular techniques generally provide greater sensitivity and specificity than conventional diagnostic methods and can facilitate the detection of low‐intensity infections and different parasite developmental stages (Jatau et al. 2016). Several ribosomal DNA regions, including ITS‐1, ITS‐2, 18S rDNA and small‐subunit ribosomal RNA, have been widely used for species identification, population genetic studies and phylogenetic analysis of *Eimeria* and other parasitic organisms (Ogedengbe et al. 2018; Tan et al. 2017; Alam et al. 2022). Although DNA extraction and amplification may be affected by the resistant nature of the oocyst wall and the presence of PCR inhibitors (Fernandez et al. 2003; Haug et al. 2007), the successful amplification obtained in this study indicates that the adopted protocols were effective.

The ITS‐1 region is particularly useful for *Eimeria* characterization because it contains conserved and variable sequences that facilitate species discrimination while also allowing assessment of intraspecific genetic variation (Alam et al. 2021; Kawahara et al. 2010; Schnitzler et al. 1999). Their multiple‐copy nature, ease of amplification, conserved flanking regions and sufficient sequence variability make them valuable markers for molecular diagnosis and phylogenetic inference, particularly among closely related taxa (Schnitzler et al. 1999; Schwarz et al. 2009). In the present study, high sequence similarity with reference strains confirmed the identity of *E. acervulina*. However, the detection of 15 genotypes and seven nucleotide substitutions indicates appreciable genetic variation within the studied population. The combination of low nucleotide diversity and relatively high genotype diversity suggests the circulation of multiple closely related variants, potentially resulting from mutation, recombination or selection pressures.

The relatively high genetic diversity observed among the *E. acervulina* sequences may partly reflect intra‐genomic variation within the ITS‐1 region rather than exclusively representing variation among parasite isolates. The nuclear ribosomal DNA array occurs in multiple copies, and sequence homogenisation among these copies through concerted evolution may be incomplete. Consequently, individual *Eimeria* isolates may contain non‐identical ITS‐1 copies, and direct PCR amplification could recover different ribosomal variants from the same genome. Such intra‐genomic polymorphism may contribute to the detection of multiple genotypes and inflate estimates of genotype and nucleotide diversity. Therefore, the 15 genotypes and seven SNPs identified among the 36 *E. acervulina* sequences may represent a combination of genuine population‐level variation and heterogeneity among ITS‐1 copies within individual parasite genomes. Sequence variation within the ITS‐1 region of chicken‐infecting *Eimeria* species has previously been reported, including differences between field‐derived sequences and corresponding reference sequences in GenBank. However, whether such variation reflects geographical differentiation, variation among isolates or heterogeneity among ribosomal copies remains difficult to determine using ITS‐1 alone (Bhaskaran et al. 2010). Studies of *E. tenella* have also identified ITS‐1 SNPs among isolates, although phylogenetic analyses indicated overall low diversity and no consistent geographical pattern (Loo et al. 2022). In the present study, the absence of clear geographical structuring among Bangladeshi *E. acervulina* genotypes could therefore reflect both parasite movement among poultry‐producing regions and the limitations associated with interpreting diversity from a multicopy ribosomal marker. Accordingly, the observed ITS‐1 variation should not be interpreted as definitive evidence of distinct circulating populations without validation using independent genetic loci or genome‐wide data.

Phylogenetic analysis provided further insight into the evolutionary relationships among the *Eimeria* isolates. The distinct separation of different *Eimeria* species and the strong clustering of *E. acervulina* isolates with corresponding reference sequences further support the suitability of ITS‐1 for phylogenetic analysis. The close association between *E. acervulina* and *E. praecox* observed in this study agrees with previous findings (Alam et al. 2022), suggesting a close evolutionary relationship between these species. Similarly, the clustering of *E. tenella* and *E. necatrix* is consistent with their recognized biological and pathogenic similarities. Comparable phylogenetic patterns have been reported in previous studies, in which closely related *Eimeria* species clustered together irrespective of their geographic origin. Within Bangladesh, the occurrence of two major *E. acervulina* clusters suggests the presence of at least two circulating genetic populations. The absence of clear geographic structuring indicates that these populations are not restricted to particular regions. This pattern may be associated with a relatively small geographic area of Bangladesh and the widespread use of commercially sourced feed, litter and other farm inputs, which may facilitate the dissemination of genetically related parasite populations between poultry‐producing regions. From a disease‐control perspective, the observed genetic variation may have important implications, because differences among parasite populations could potentially influence virulence, host immune responses and susceptibility to anticoccidial drugs or vaccines. Therefore, continued molecular surveillance of *E. acervulina* populations is important for understanding their population structure and for developing more effective and sustainable strategies for coccidiosis control.

### 4.1 Study Limitations

This study has several limitations. First, genetic diversity and phylogenetic analyses were based exclusively on the ITS‐1 region. Although ITS‐1 is useful for molecular identification of *Eimeria* species because of its sequence variability, it is a multicopy ribosomal marker and may contain intra‐genomic variation. Consequently, differences among ITS‐1 sequences may not always represent true genetic differentiation among *E. acervulina* isolates. Reliance on a single locus also means that the resulting phylogeny reflects the evolutionary history of ITS‐1 rather than that of the complete parasite genome. The identification of two phylogenetic clusters should therefore be interpreted cautiously and should not, by itself, be considered conclusive evidence of two genetically discrete *E. acervulina* populations. Second, only 36 PCR‐positive samples were sequenced, although samples were collected from seven major poultry‐producing regions of Bangladesh. This subset may not capture the complete genetic diversity of *E. acervulina* circulating throughout the country. Differences in the number of samples obtained from individual regions, farms, production systems or chicken types may also have influenced genotype frequencies and the apparent absence of geographical structuring. Furthermore, samples originated from commercial broiler and Sonali farms operating under floor‐rearing systems; therefore, the findings may not be fully representative of backyard poultry, layer farms, breeder flocks or other management systems. Future studies should include larger and more geographically balanced sample sets and incorporate independent single‐copy nuclear markers, mitochondrial loci, antigen‐associated genes or whole‐genome sequencing. Analysing multiple molecular markers would help distinguish intra‐genomic ITS‐1 polymorphism from genuine variation among isolates and provide more robust evidence of the population structure and geographical distribution of *E. acervulina* in Bangladesh.

## 5 Conclusion

This study provides the first comprehensive molecular characterization and phylogenetic analysis of *E. acervulina* isolates from poultry in Bangladesh using the ITS‐1 gene. A high prevalence of *E. acervulina* confirmed its dominant role in chicken coccidiosis in the studied regions. Phylogenetic analysis demonstrated clear species‐level clustering and revealed the presence of two genetically distinct populations circulating in Bangladesh. These findings highlight the utility of ITS‐1 as a reliable molecular marker for species identification and genetic analysis of *E. acervulina*. Overall, this study establishes a foundation for future investigations employing higher‐resolution approaches, such as multi‐locus sequence typing (MLST) or whole‐genome analysis, to support improved epidemiological monitoring, vaccine development and control strategies for coccidiosis in poultry.

## Author Contributions

**Bimal Chandra Karmakar**: introduction, methodology, investigation, software, data curation, data analysis, writing – review and editing, writing – original draft, visualization. **Nusrat Nowrin Shohana**: methodology, writing – original draft, writing – review and editing, software. **Subir Sarker**: software, writing – review and editing. **Anita Rani Dey**: writing – review and editing, formal analysis, software, validation, supervision. **Anisuzzaman Anisuzzaman**: validation, formal analysis, supervision, writing – review and editing. **Sharmin Aqter Rony**: methodology, funding acquisition, writing – review and editing. **Shanaz Parvin**: methodology, writing – review and editing. **Saykat Kumar Debnath**: methodology, writing – review and editing. **Shirin Akter**: methodology, writing – review and editing. **Mohammad Zahangir Alam**: conceptualization, methodology, supervision, funding acquisition, writing – review and editing, validation, project administration, resources, visualization.

## Funding

This work was supported by BAS‐USDA (Project number: LS‐06, 5th phase).

## Ethics Statement

The study was approved by the Animal Welfare and Experimentation Ethics Committee of Bangladesh Agricultural University, Mymensingh (Approval number: AWEEC/BAU/2023 (46)).

## Conflicts of Interest

The authors declare no conflicts of interest.

## Data Availability

The data that support the findings of this study are available from the corresponding author upon reasonable request.
